# Modeling and Regulation of Dynamic Temperature for Layer Houses Under Combined Positive- and Negative-Pressure Ventilation

**DOI:** 10.3390/ani14213055

**Published:** 2024-10-23

**Authors:** Lihua Li, Min Li, Yao Yu, Yuchen Jia, Zhengkai Qian, Zongkui Xie

**Affiliations:** 1 College of Mechanical and Electrical Engineering, Hebei Agricultural University, Baoding 071000, China; llh@hebau.edu.cn (L.L.); 20227090976@pgs.hebau.edu.cn (M.L.); younger1981@hebau.edu.cn (Y.Y.); jiayuchen1981@163.com (Y.J.); 20237091033@pgs.hebau.edu.cn (Z.Q.); 2 Key Laboratory of Broiler/Layer Breeding Facilities Engineering, Ministry of Agriculture and Rural Affairs, Baoding 071000, China; 3 Hebei Provincial Key Laboratory of Livestock and Poultry Breeding Intelligent Equipment and New Energy Utilization, Baoding 071000, China

**Keywords:** layer houses, combined positive- and negative-pressure ventilation, energy and mass balance, temperature simulation, fuzzy PID control

## Abstract

The environmental control of layer houses with multi-tiered cage systems is influenced by factors such as the structure of the henhouses and the heat dissipation of the flock, leading to low precision and large fluctuations in temperature control. Based on a new combined positive- and negative-pressure ventilation (CPNPV) mode, a dynamic temperature model is constructed. Additionally, a temperature control method for a layer house is designed using a variable universe fuzzy PID control algorithm (VFPID). First, based on the principles of energy and mass balance, and by decoupling the relationship between positive- and negative-pressure ventilation volumes, a dynamic temperature model for layer houses under CPNPV was established. Then, the PID parameters and the proportional relationship between positive- and negative-pressure ventilation were optimized through fuzzy rules, and a proportional exponential function was introduced to adjust the scaling of the universe, enabling fine-tuned control. Finally, a temperature control model for the layer house was built using Simulink. The results show that the coefficients of determination (R^2^) of the constructed dynamic temperature models are between 0.79 and 0.88, respectively, indicating high accuracy. The designed VFPID method outperformed traditional on–off control and improved control precision by 20–23.53% and 10.34–22.22% compared with PID control and fuzzy PID(FPID) control methods, respectively. This study provides new insights for the development of environmental control equipment and precise environmental regulation of layer houses.

## Simple Summary

Maintaining a stable and precise temperature in poultry houses is essential for the well-being and productivity of layer hens. However, traditional methods of temperature control often face challenges due to the complex structure of henhouses and the heat produced by the birds themselves. This study investigates a novel positive–negative pressure ventilation system for improved temperature control in multi-tier layer houses. A dynamic model is developed to better understand the temperature changes under this new system, decoupling the coupling relationship between positive and negative pressure ventilation volumes. Furthermore, a variable universe fuzzy PID is designed to cooperatively control the ventilation rate of positive and negative pressure fans to ensure accurate temperature regulation for henhouses. Simulation and experimental results show that this approach significantly outperforms traditional control methods, offering more precise temperature management, which can lead to healthier layer hens and potentially higher egg production. This research contributes valuable insights to the development of advanced environmental control systems in the poultry industry, aiming to create better living conditions for the birds and improve overall farm efficiency.

## 1. Introduction

With the scale and intensification of the layer farming industry, environmental control within henhouses has become increasingly crucial. An efficient environmental control system can promote the healthy growth of layer hens, enhance egg production and quality, reduce disease incidence, and lower mortality rates [1]. However, environmental control equipment in multi-layered cage farming henhouses typically employs on–off control, which struggles to achieve precise and stable control of environmental parameters in high-temperature conditions. Particularly during hot summer weather, inadequate ventilation can lead to excessively high temperatures, causing heat stress in layer hens and consequently affecting their welfare and productivity [2,3]. Therefore, studying stable and efficient methods for regulating the environment of henhouses is crucial for achieving precise control over the conditions inside.

Exploring the multiple influencing factors of environmental parameters in livestock and poultry houses and their coupling laws is the foundation for precise environmental control. Currently, researchers have developed various models and algorithms to establish environmental models for predicting conditions in livestock and poultry houses. Gautam et al. [4] and Hyeon et al. [5] used CFD models to evaluate the impact of different ventilation system designs on the internal environment of pig houses. Thompson et al. [6] developed a dynamic mechanistic heat balance model to accurately predict the heat balance and physiological responses of cattle under changing weather conditions by analyzing their physiological responses and external environmental conditions. However, the complexity of this model hinders its practical application. Liang Chao et al. [7] and Er Mengwei et al. [8] established an hourly dynamic prediction model for layer houses and a microclimate environment model for brooding houses, respectively, with average indoor temperature prediction errors between 0.67 and 1.3 °C, demonstrating high accuracy. Building on this, Wang Lijie et al. [9] developed models for harmful gases in livestock and poultry houses, with R^2^ values all above 0.84. These models, based on principles of physics, mathematics, and biology, and extensive experimental data, simulate indoor temperatures. Additionally, some models utilize machine learning and deep learning methods such as long short-term memory neural networks, fuzzy clustering, and adaptive neuro-fuzzy inference systems (ANFIS) [10,11], all capable of accurately predicting environmental conditions in livestock and poultry houses. The above studies mainly focus on indoor temperature, humidity, and harmful gas models under negative-pressure ventilation. However, the literature [12,13] indicates that while pure negative-pressure ventilation systems effectively control local pollution, they are limited in improving overall air quality. The literature [14,15,16] suggests that CPNPV can effectively improve the uniformity of airflow within henhouses, maintain suitable thermal environmental parameters, and promote poultry health. Additionally, CPNPV is commonly used in hospital operating rooms and wards to minimize cross-contamination [17]. However, there is limited discussion on CPNPV, and no studies have been conducted on constructing temperature models for livestock and poultry houses under this combined ventilation mode.

In terms of environmental control, as technology advances and the need for precise control increases, control methods have become increasingly important. Fairchild [18] pointed out that modern broiler house environmental control systems are highly dependent on electronic controllers, which can precisely regulate temperature and humidity to maximize the production potential of broilers. Lahlouh et al. [19] developed a novel Multiple-Input Multiple-Output (MIMO) fuzzy PID controller, achieving precise control of temperature, humidity, and ammonia concentration in livestock and poultry houses, allowing the system to operate stably in complex environments and effectively improving indoor environmental quality. Tan Zichao et al. [20], and Zhang et al. [21] developed real-time environmental monitoring systems for livestock and poultry houses, which optimize indoor temperature, humidity, ventilation, and harmful gas concentrations using precise control technology, significantly improving production efficiency and animal welfare. Barros et al. [22] showed that applying PID control in pig houses, compared with traditional thermostats, achieves a more uniform air temperature distribution, effectively improving the thermal comfort of pigs. Researchers Phu et al. [23] significantly improved control accuracy by optimizing PID parameters using intelligent control algorithms. Lahlouh et al. [24] designed and implemented a state-PID feedback controller for poultry systems, achieving better temperature and humidity control under winter climate conditions. However, the aforementioned studies primarily focus on environmental control in livestock and poultry houses under a single negative-pressure ventilation mode. The CPNPV mode presents greater control challenges due to the coupling of ventilation volumes between positive- and negative-pressure fans, rendering the previously mentioned methods unsuitable. Therefore, it is necessary to further develop efficient and intelligent control methods specifically designed for environmental control systems utilizing CPNPV.

To address the above issues, this paper constructs a dynamic temperature model for layer houses under CPNPV mode and designs a temperature control method for henhouses using a variable universe fuzzy PID control algorithm (VFPID) to achieve precise environmental regulation in layer houses. The innovative contributions of this paper are as follows:

(1) First, based on the principles of energy and mass balance, a dynamic temperature model for layer houses under CPNPV mode is established by decoupling the ventilation volumes of positive- and negative-pressure fans.

(2) Then, utilizing the constructed dynamic temperature model, the VFPID method adjusts the ventilation volumes of positive- and negative-pressure fans to regulate the indoor temperature. The PID parameters and the proportional relationship of positive- and negative-pressure ventilation volumes are optimized through fuzzy rules, and a proportional exponential function is introduced to adjust the scaling of the universe.

(3) Finally, a temperature control model for the layer house is built using Simulink. The dynamic temperature model and control method are simulated and verified the for summer, autumn, and winter seasons.

## 2. Construction of Dynamic Temperature Model for Layer House

This chapter constructs a dynamic temperature model for a layer house under CPNPV based on the principles of energy and mass balance, as well as actual monitored environmental data from the layer house.

### 2.1. Heat Balance Analysis of Layer House

**Figure 1:**
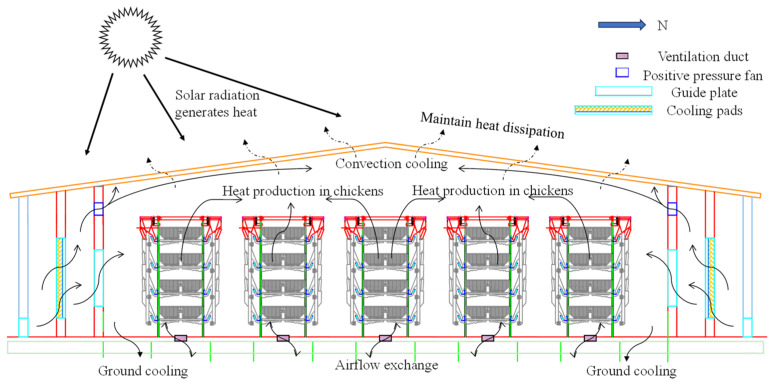
Schematic diagram of heat exchange in a layer house under CPNPV.

The input data for the dynamic temperature model of the layer house include external environmental parameters (temperature, humidity, atmospheric pressure, solar radiation), internal environmental parameters (temperature, humidity, atmospheric pressure), total weight of the flock, daily egg production, total ventilation volume per minute, and the thermal conductivity of the building. The output is the indoor temperature. The heat exchange mechanism within the henhouse is shown in Figure 1. The process for calculating the model is as follows: First, the input data are processed through the energy and mass balance module to calculate the heat production and dissipation in each component. Then, these heat quantities are algebraically summed, and the total heat inside the henhouse is integrated to solve for the indoor temperature.

Based on the environmental conditions within the henhouse, the heat exchange equation for caged layer houses [25] is shown in Equation (1).

(1) $$\rho Vc_{p}\frac{\partial T_{i}}{\partial t}=Q_{s}+Q_{c}+Q_{o}-Q_{v}-Q_{g}-Q_{w}-Q_{a}$$

where ρ is air density, in kg/m^3^; *V* is the volume of the henhouse, in m^3^; $c_{p}$ is the specific heat capacity of air in the henhouse, in J/(kg °C); $T_{i}$ is the temperature inside the henhouse, in °C; $\partial T_{i}/\partial t$ is the rate of temperature change inside the house, in °C/s; $Q_{s}$ is the heat gained from solar radiation through the roof per unit time, in W; $Q_{c}$ is the sensible heat produced by layer hens per unit time, in W; $Q_{o}$ is the sum of heat gained from lighting, electrical equipment, etc. in the henhouse, in W; $Q_{v}$ is the heat lost through ventilation in the henhouse per unit time, in W; $Q_{g}$ is the heat lost through the floor in the henhouse per unit time, in W; $Q_{w}$ is the heat lost through the maintenance structure in the henhouse per unit time, in W; and $Q_{a}$ is the heat dissipated through tiny gaps, in W. Since the heat gained ($Q_{o}$) from lighting and electrical equipment, as well as the heat dissipated ($Q_{a}$) through tiny gaps, is very small, it can be neglected. By integrating Equation (1), the simulated temperature inside the henhouse is obtained.

The heat produced by solar radiation $Q_{s}$ is calculated as shown in Equation (2).

(2) $$Q_{s}=P_{s}S_{w}I$$

where $P_{s}$ is the radiation conversion coefficient of the enclosure structure; $S_{w}$ is the effective area of the enclosure structure receiving solar radiation, in m^2^; and I is the solar irradiance intensity, in W/m^2^.

To calculate the sensible heat gained from the layer hens’ body surfaces $Q_{c}$, we first calculate the total heat produced by layer hens $Q_{t}$. According to the International Commission of Agricultural and Biosystems Engineering (CIGR) guidelines [26], it is calculated as shown in Equation (3).

(3) $$Q_{t}=(6.28M^{0.75}+25Y)(4\times 10^{-5}{\left(20-T_{i}\right)}^{3}+1)$$

where M is the total weight of the layer hens, in kg, and Y is the total egg production, in kg/d. The relationship between $Q_{c}$ and $Q_{t}$ is shown in Equation (4).

(4) $$\frac{Q_{c}}{Q_{t}}=0.67(1-0.02(20-T_{i}))-9.8\times 10^{-11}T_{i}^{6}$$

The calculation of heat lost through ventilation $Q_{v}$ is detailed in Section 2.2, the model of heat loss under CPNPV.

The heat lost $Q_{g}$ due to convective heat exchange between the floor and indoor air is calculated as shown in Equation (5).

(5) $$Q_{g}=h_{g}S_{g}(T_{i}-t_{g})$$

where $h_{g}$ is the heat transfer coefficient of the indoor floor, in W/(m^2^ °C); $S_{g}$ is the surface area of the indoor floor, in m^2^; and $t_{g}$ is the temperature of the indoor floor, in °C, which can be substituted by the manure pit temperature.

The heat dissipation of the indoor enclosure structure $Q_{w}$ is calculated using the thermal load recommended by the “Design Code for Heating Ventilation and Air Conditioning of Industrial Buildings” [27], as shown in Equation (6).

(6) $$Q_{w}=k_{s}F_{s}(t_{n}-t_{w})$$

where $k_{s}$ is the heat transfer coefficient of the enclosure structure; $F_{s}$ is the surface area of the henhouse enclosure structure, in m^2^; $t_{n}$ is the temperature of the inner surface of the enclosure structure, in °C; and $t_{w}$ is the temperature of the outer surface of the enclosure structure, in °C.

**Remark** **1.** *Figure 1* *shows the ventilation duct located 1 m below the ground, which extends underground for a certain distance before gradually rising to the surface. Inside the hen house, the end of the duct extends approximately 10 cm above the ground; outside the hen house, the end of the duct is positioned at a height of 1.8 m above the ground. In the process of model construction, this paper simplifies the heat transfer process of building enclosure structures, assuming it as heat transfer of homogeneous multi-layer materials under the same conditions, ignoring secondary influencing factors such as contact thermal resistance, flowing air interlayer, and roof slope [27]. This simplification allows the model to focus on the main heat transfer mechanisms, improving the processing speed of the model while maintaining accuracy*.

### 2.2. Modeling of Heat Loss Under CPNPV

The experimental henhouse adopts a new ventilation strategy, namely a CPNPV system. This system regulates the ventilation volume inside the house through the synergistic effect of positive- and negative-pressure ventilation. Whether it is positive-pressure active ventilation or negative-pressure passive ventilation, all air entering the house needs to be cooled through cooling pad. Through this combined ventilation method, the house can achieve good temperature control and air circulation, effectively improving the environmental quality inside the house.

The heat lost through ventilation $Q_{v}$ is calculated as shown in Equation (7).

(7) $$Q_{v}=mc_{p}(T_{i}-t_{c})$$

where $t_{c}$ is the temperature of the air after cooling through the cooling pad. This parameter is only applicable when the cooling pad is open, calculated as shown in Equation (8). When the cooling pad is not open, it is equal to the outdoor air dry-bulb temperature $t_{0}$ [7].

(8) $$t_{c}=t_{s}+(t_{0}-t_{s})exp(-12.28305\nu^{-0.30078}\frac{H}{\rho c_{p}})$$

where $t_{s}$ is the outdoor air wet-bulb temperature, in °C; ν is the air speed through the cooling pad, in m/s; *H* is the thickness of the cooling pad, in m; and *m* is the mass flow rate of ventilation in the house, in kg/s, calculated as shown in Equation (9).

(9) m=ρL

where L is the total ventilation volume, in m^3^/s, calculated as

(10) L=AV

where *A* is the area of the fan or ventilation opening, in m^2^, and *V* is the air speed at the fan outlet or in front of the cooling pad, in m/s.

The measurement of *L* has two methods: measuring the total ventilation volume at the air inlet or measuring the total ventilation volume at the exhaust outlet. After a certain period, the inlet and outlet air volumes will reach a balance, as shown in Equation (11), which can reflect the relationship between these two.

(11) $$L_{-}=L_{d}+L_{+}$$

where $L_{-}$ is the negative-pressure ventilation volume, $L_{d}$ is the ventilation volume at the guide plate, and $L_{+}$ is the positive-pressure ventilation volume. Both $L_{d}$ and $L_{+}$ are inlet ventilation volumes, used to send air cooled by the cooling pad into the house to regulate the indoor temperature. The exhaust outlet is equipped only with negative-pressure fans, which are responsible for cooling and discharging pollutants.

In practice, the CPNPV mode will maintain the house in a slight negative-pressure state, this pressure difference ensures a continuous unidirectional flow of external air into the enclosure, preventing airflow backflow. In addition, the guide plate can automatically open or close, where $L_{+}$ is slightly smaller than $L_{-}$. The relationship between positive- and negative-pressure fan ventilation volumes is shown in Equation (12).

(12) $$L_{+}=\lambda L_{-}$$

where λ is a variable, with a value range of [0, 1], which λ reflects the proportion relationship between positive- and negative-pressure ventilation volumes in the house ventilation process.

It is important to note that the guide plate is not fully opened to 90°. In fact, the angle between the guide plate and the wall is about 10–20°. In this case, the airflow entering the guide plate mainly comes from the central position of the lower part of the cooling pad. Additionally, the positive-pressure fan faces away from the outer wall, so the airflow does not enter perpendicularly. When both the positive- and negative-pressure fans are operating simultaneously, two streams of airflow form within the compartment: one part of the air enters the positive-pressure fan, while the other part enters the guide plate. The air from the lower part of the cooling pad is entirely drawn into the interior by the negative-pressure fan, while the airflow from the upper part of the cooling pad can only enter the interior through the positive-pressure fan.

Considering the actual situation described above, necessary simplifications and reasonable assumptions were made during the modeling process. Therefore, the air passing through the cooling pad is divided into two parts: the upper part enters the positive-pressure fan, and the lower part enters the guide plate. Due to the influence of the positive-pressure fan, there is a difference in air speed between the upper and lower parts of the cooling pad. Therefore, the calculation of ventilation heat dissipation can be decomposed into two parts: heat dissipation of positive-pressure fan $Q_{v+}$ and heat dissipation at the guide plate $Q_{vd}$. According to Equation (8), to calculate the temperature $t_{c}$ after passing through the cooling pad, it is necessary to determine the air speed v through the cooling pad. The air speed in the upper half of the cooling pad can be approximated to the air speed of the positive-pressure fan $v_{+}$, while the air speed in the lower half can be approximated to the air speed at the guide plate $v_{g}$. There is a 1.5-meter wide compartment between the cooling pad and the positive-pressure fan and guide plate. Due to the narrow distance of the compartment, the air speed attenuation is small, so it can be reasonably assumed that the air speed through the upper and lower parts of the cooling pad remains basically unchanged. This assumption simplifies the problem analysis and facilitates subsequent quantitative calculations of the cooling pad cooling process.

Therefore, for the positive-pressure ventilation heat dissipation $Q_{v+}$ and guide plate ventilation heat dissipation $Q_{vd}$, their ventilation mass flow rates are $m_{+}$ and $m_{d}$, respectively. The air temperatures after cooling through the cooling pads are $t_{c+}$ and $t_{cd}$, respectively, and the air speeds through the cooling pads are $v_{+}$ and $v_{d}$, respectively. These parameters can be substituted into Equation (7) for heat dissipation calculation, Equation (8) for calculating air temperature after cooling through the cooling pad, and Equation (9) for determining the ventilation mass flow rate in the house, thereby obtaining the respective values for each process. Here, $v_{+}$ and $v_{d}$ can be solved by combining Equations (10)–(12), calculated as shown in Equations (13) and (14).

(13) $$v_{+}=\frac{\lambda L_{-}}{A_{+}}$$

(14) $$v_{d}=\frac{(1-\lambda )L_{-}}{A_{d}}$$

where $A_{+}$ is the sum of the areas of all positive-pressure fans, and $A_{d}$ is the sum of the cross-sectional areas of all guide plates.

In summary, the heat dissipation of positive- and negative-pressure combined ventilation $Q_{v}$ is calculated as shown in Equation (15).

(15) $$Q_{v}=Q_{v+}+Q_{vd}$$

**Remark** **2.** *The guide plate primarily serves two purposes: 1. It adjusts the direction and speed of the air entering from outside the coop, which can optimize air circulation inside and improve the ventilation effect in the chicken coop. 2. By reasonably adjusting the angle of the baffle, it prevents the cold air cooled by the cooling pad from blowing directly onto the chickens, thereby preventing them from catching cold*.

The model in this paper follows the actual situation to set the cooling pad opening conditions, based on the measured data from different seasons to set the opening state of the cooling pad. The data from two days in summer show that the cooling pad is in an open state, while the data from four days in autumn and winter show that the cooling pad is closed. Therefore, in the model simulation of this paper, referring to the measured situation, the cooling pad is set to be open in summer and closed in autumn and winter.

### 2.3. Data Sources and Monitoring

The data used in this study were obtained from a layer farm in Shahe City, Hebei Province, China. The farm employs a multi-tiered cage rearing system with a CPNPV mode. The henhouse has a total of 5 rows and 4 tiers, with cages on both sides of each row, housing a total of 48,000 white-feathered layer hens. The henhouse is oriented east-west, with a length of 100 m, a width of 15 m, a height of 4 m, and a ridge height of 4.5 m.

Eighteen negative-pressure fans (model EM50, Shandong Mumin Machinery Co., Ltd., Qingdao, Shandong Province, China) are installed on the east wall, arranged in two rows of nine fans each. These fans primarily facilitate longitudinal ventilation to achieve pollutant removal and cooling. The vertical spacing between the two rows of fans is 40 cm, and the horizontal spacing is 20 cm, forming a uniform ventilation layout.

**Figure 2:**
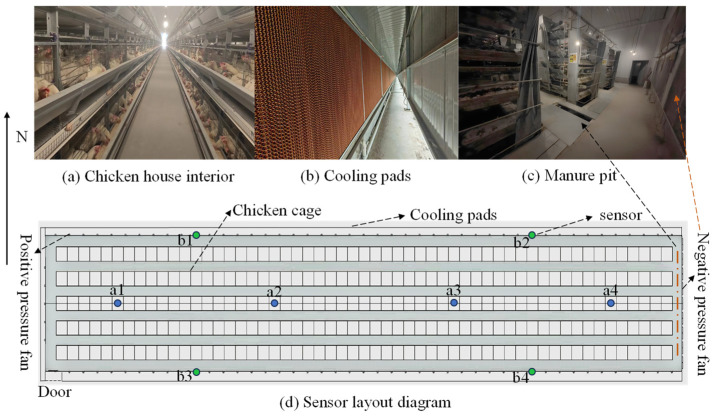
The actual layout of the layer house and the placement of sensor locations.

Ninety-five positive-pressure fans (model YWF2D-300, Taizhou Qinlang Electromechanical Co., Ltd., Wenling, Zhejiang Province, China) are installed in the henhouse to simulate natural wind, providing a degree of cooling. These fans are evenly distributed on the south wall, north wall, and gable walls, with 45 fans on each of the south and north walls, and 5 fans on the gable walls. Cooling pads are installed between the outer wall insulation board and the guide plate, with each set of cooling pads having a thickness of 15 cm, a height of 2.13 m, and a length of 21 m. Figure 2a illustrates the overall structure of the hen house; Figure 2b shows a real image of the single-side cooling pad; and Figure 2c marks the specific location of the manure pit, which is positioned in front of the negative-pressure fan.

This study utilized multiple sensors to accurately measure various environmental parameters within the henhouse. The sensor parameters are shown in Table 1.

In terms of sensor arrangement, sensors are spaced 25 m apart along the length of the hen house to capture environmental differences between the front and rear ends. Vertically, considering that the hen house consists of four layers of cages, the sensors are installed on the second layer of cages. This height enables adequate monitoring of temperature variations between the upper and lower layers while also maximizing the representation of the overall heat production of the flock. To avoid interference from localized environmental factors, the sensors are deliberately positioned away from areas such as ventilation openings that may affect measurement accuracy. As shown in Figure 2d, the hen house is evenly divided into four regions: a1, a2, a3, and a4. Two sensors are installed at the central location of each region, one for measuring temperature and humidity (model: RS-WS-2D) and the other for measuring atmospheric pressure (model: RS-QY-2-4). All these sensors are mounted at the central position of the second layer of cages at a height of 1.65 m above the ground.

The surface temperatures of the inner and outer walls were measured using infrared temperature sensors (RS-WD-HW-I20), as shown at points b1, b2, b3, and b4 in Figure 2d. Two sensors were placed in a mirrored position inside and outside the layer house, fixed 40 cm from the south (north) wall and 2 m above the ground. This arrangement allows for monitoring the temperature distribution on different walls within the hen house. By comparing the temperature differences between the inner and outer wall surfaces, the heat loss through the walls can be calculated. At a height of approximately 2 m outside the layer house, temperature, humidity, and atmospheric pressure sensors were installed to monitor the external environmental parameters. All data collected inside and outside the layer house were recorded at 1-min intervals.

Air speed is measured using thermal anemometers (model GT8911), placed 1 m in front of the large fan outlets and 20 cm in front of the small fan inlets, ensuring that the probes are centered within the airflow [28]. Before the measurements, we calibrated the anemometer to ensure the accuracy of the results. Ventilation volume is calculated by continuously monitoring the operating time, air speed, and activation status of the fans, with the operational data exportable from the environmental control equipment.

Solar radiation intensity data are sourced from historical reanalysis datasets provided by the European Centre for Medium-Range Weather Forecasts (ECMWF)/National Aeronautics and Space Administration (NASA), accessed via www.xihe-energy.com (accessed on 1 March 2024) [29]. The data, initially available at 1-hour intervals, are processed using linear interpolation to yield data at 1-min intervals. Partial environmental data are presented in Table 2.

## 3. VFPID-Based Temperature Controller

**Figure 3:**
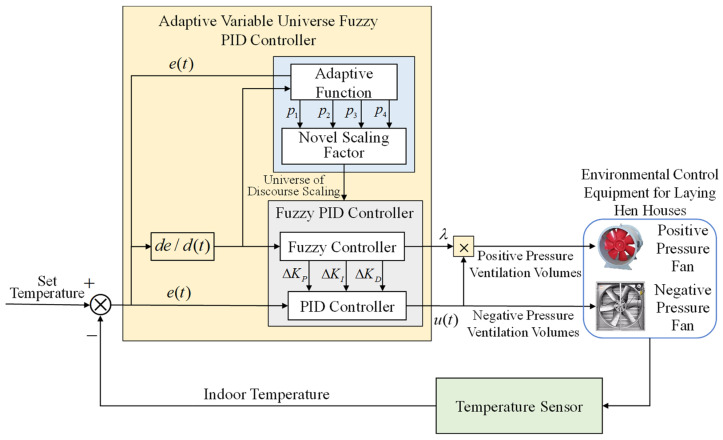
Temperature control schematic of the layer house: *e*(*t*) is the error between the actual temperature and the set temperature. *de/d*(*t*) is the rate of change in the error between the simulated temperature and the set temperature. *P*_1_, *P*_2_, *P*_3_, *P*_4_ are scaling factors. $\Delta K_{P},\;\Delta K_{I}$, and $\Delta K_{D}$ represents the correction values for PID coefficients. λ is the ratio coefficient between positive- and negative-pressure ventilation volumes. *u*(*t*) is the negative-pressure ventilation volume output by the controller.

In this section, based on the aforementioned heat exchange dynamic temperature model, a VFPID control method is adopted to control the temperature inside the layer house. By introducing fuzzy logic, this method not only determines the coupling relationship between positive- and negative-pressure ventilation volumes but also enhances the flexibility and robustness of the traditional PID control method, making temperature control more precise and stable. This method analyzes temperature control deviation and automatically adjusts PID parameters, as well as the ratio of positive- to negative-pressure ventilation volumes, effectively coping with the complex changes in the henhouse environment to ensure constant indoor temperature, providing an ideal growth environment for the layer hens. The control structure of the VFPID-based method for the layer house is shown in Figure 3. The control structure and parameters presented in Figure 3 are explained in detail in the following subsections.

### 3.1. Fuzzy PID Controller Based on CPNPV

In traditional PID controllers, $K_{P},\;K_{I}$ and $K_{D}$ represent the proportional, integral, and derivative coefficients, respectively, which can be adjusted to optimize the controller’s stability and response speed. Numerous case studies and experiments have demonstrated that using fixed $K_{P},K_{I}$ and $K_{D}$ coefficients fails to adapt to actual environmental conditions, resulting in suboptimal control effects [30]. Furthermore, in the CPNPV mode, λ in Equation (12) is used to determine the ratio of positive- to negative-pressure ventilation volumes. In practice, λ may fluctuate due to changes in external environmental conditions. Therefore, treating λ as an adjustable parameter is crucial for accurately calculating the heat dissipation through ventilation inside the henhouse.

To address these issues, this study combines fuzzy control with PID control, designing a dual-input, quad-output fuzzy PID(FPID) control structure. The input variables are the error e(t) between the set temperature and the actual temperature, and its rate of change ec(t). The output variables are the correction values for the PID coefficients ($\Delta K_{P},\Delta K_{I},\Delta K_{D}$) and the ratio coefficient λ between positive- and negative-pressure ventilation volumes. Through fuzzy processing, fuzzy inference, and decision logic, the controller dynamically adjusts the correction values of PID control parameters, modifying PID parameters in real time [31] to enhance control precision. The controller output u(t) is expressed as shown in Equation (16).

(16) $$u(t)=(K_{P}+\Delta K_{P})e(t)+(K_{I}+\Delta K_{I})\int_{0}^{t}e(t)dt+\;(K_{D}+\Delta K_{D})\frac{de(t)}{dt}$$

For different temperature errors e(t) and error change rates ec(t), the adjustment of controller parameters $K_{P},K_{I},K_{D}$, and λ should follow these principles to ensure optimized performance:

(1) When e(t) is large, the proportional gain $K_{P}$ should be increased to accelerate system response, while the derivative gain $K_{D}$ should be reduced to enhance the system’s resistance to external disturbances. The system exhibits good tracking performance in this state. Additionally, to reduce system overshoot, the integral gain $K_{I}$ should be appropriately decreased.

(2) When e(t) is moderate, a smaller $K_{P}$ should be selected to minimize system overshoot, while $K_{I}$ and $K_{D}$ should be adjusted to appropriate values. This helps balance rapid response and excessive adjustment, achieving stable control.

(3) When e(t) is small, parameter adjustment primarily aims to reduce steady-state error and control the error change rate ec(t). Therefore, $K_{P}$ and $K_{I}$ should be set to larger values to ensure good steady-state performance. Simultaneously, $K_{D}$ should be adjusted according to the changing trend of ec(t) to prevent system oscillation.

(4) The selection of λ prioritizes e(t). When e(t) is negative (temperature above the set value), a larger λ should be chosen to increase positive-pressure ventilation for cooling. When e(t) is positive (temperature below the set value), a smaller λ should be selected to reduce positive-pressure ventilation and maintain indoor temperature.

Based on the practical requirements of henhouse temperature control and expert experience, fuzzy control rules were established, as shown in Table 3. The fuzzy linguistic variables for e(t) and ec(t) are defined as “NB (Negative Big), NM (Negative Medium), NS (Negative Small), ZO (Zero), PS (Positive Small), PM (Positive Medium), PB (Positive Big)”, each containing seven fuzzy subsets, resulting in a total of 49 fuzzy rules [32]. The initial universe of discourse for e(t) is $\left[-E_{1},E_{1}\right]$, for ec(t) is $\left[-E_{2},E_{2}\right]$, and for the output variable U is $\left[-U_{3},U_{3}\right]$. In the model, a compensation coefficient λ is introduced to adjust the positive-pressure ventilation rate $L_{+}$ in real time. The value of λ is controlled by a fuzzy controller based on different values of e(t) and ec(t), with the initial domain of [0, 1]. The initial domain of e(t) is set to [−3, 3], and the initial domain of ec(t) is set to [−1.5, 1.5]. The output variable *U* has an initial domain of [−5, 5].

**Remark** **3.** *The VFPID-based temperature control model for layer houses primarily consists of two parts: the controller and the controlled system. The controller adjusts the positive- and negative-pressure ventilation volumes (output variables) of the controlled system based on e(t)* *and ec(t)* *between the set temperature and actual temperature. The ventilation rate then serves as an input to the indoor dynamic temperature model established in Section 2, with the model’s output feeding back to the temperature controller, forming a closed-loop control system. The model adjusts the upper and lower limits of ventilation volumes according to seasonal changes to prevent deviation from actual conditions and maintain model accuracy. Based on conclusions from literature [33,34], the maximum ventilation rate required in summer is approximately 0.08 $m^{3}/(\min\cdot \mathrm{kg})$.* *Therefore, the simulation sets the maximum indoor ventilation rate not to exceed 7435 $m^{3}/\min$.* *In winter, the minimum ventilation rate required for lay* *er hens is about 0.02 $m^{3}/(\min\cdot \mathrm{kg})$,* *so the simulation sets the minimum ventilation rate not to fall below 1858 $m^{3}/\min$*.

### 3.2. Variable Universe Expansion Factors

To further enhance the control performance of the fuzzy PID, scaling factors are introduced to adjust the universe of discourse ranges for input and output variables in the fuzzy controller online, further optimizing the PID parameter adjustment strategy. Additionally, a compensation coefficient λ is introduced in the model to adjust the positive-pressure ventilation volume $L_{+}$. The fuzzy controller controls λ based on different values of parameters e(t) and ec(t), with an initial universe of discourse for λ set to [0, 1].

**Figure 4:**
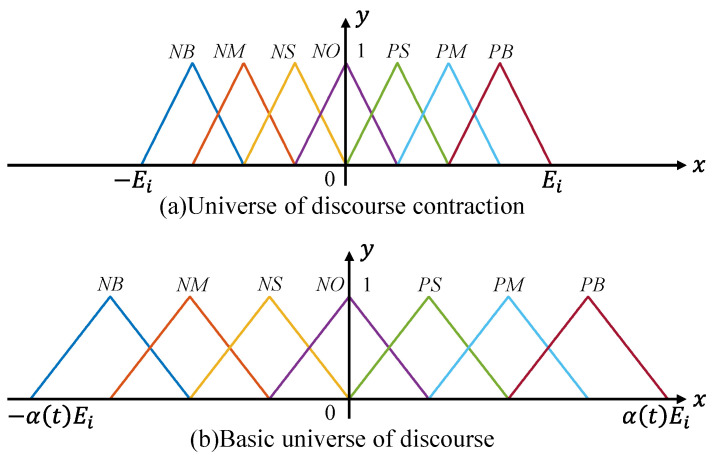
Basic principle of variable universe of discourse.

After introducing the variable domain concept, the variable domain adjustment is shown in Equation (17).

(17) $$\left\{\begin{array}{l} X_{e}=\left[-\alpha \left(e\left(t\right)\right)E_{1},\alpha \left(e\left(t\right)\right)E_{1}\right]\\ X_{\Delta e}=[-\alpha (\Delta e(t))E_{2},\alpha (\Delta e(t))E_{2}]\\ Y_{U}=[-\beta (e(t),\Delta e(t))U_{3},\beta (e(t),\Delta e(t))U_{3}] \end{array}\right.$$

where α and β are the scaling factors to be determined, and $X_{e}$, $X_{\Delta e}$, and $Y_{U}$ are the new universes of discourse (shown in Figure 4a) obtained after scaling transformation of the basic universes of discourse e(t), ec(t), and U (Figure 4b), respectively.

The scaling factors are reasonably adjusted according to the magnitude of the temperature error: When the temperature error is large, a detailed division of fuzzy control rules is unnecessary, and the universe of discourse remains unchanged to avoid contraction or expansion. When the temperature error is moderate, simple fuzzy rules may not provide ideal control effects, so the universe of discourse is moderately reduced to refine control rules and enhance control precision. As the temperature error gradually approaches zero, the universe of discourse should be rapidly reduced to make control rule divisions more detailed, achieving precise control over extremely small temperature differences. The scaling factors employ special functions, effectively avoiding potential control performance degradation issues that may arise from fuzzy rule-based methods. This paper adopts a proportional exponential function as the scaling factor [35], as shown in Equation (18).

(18) $$\left\{\begin{array}{l} \alpha (e(t))={\left(\frac{\left|e(t)\right|}{E_{1}}\right)}^{P_{1}}+\theta \\ \alpha (ec(t))={\left(\frac{\left|ec(t)\right|}{E_{2}}\right)}^{P_{2}}+\theta \\ \beta (e(t),\Delta e(t))=\frac{{\left(\frac{\left|e(t)\right|}{E_{1}}\right)}^{P_{3}}+{\left(\frac{\left|ec(t)\right|}{E_{2}}\right)}^{P_{4}}}{2} \end{array}\right.$$

where $P_{i}$ is scaling factor, with $P_{i}$ ∈ [0, 1], i = 1, 2, 3, 4. To avoid zero domain boundaries caused by zero values of parameters α(e(t)) and α(ec(t)), θ is set as a sufficiently small positive number.

It should be noted that in existing research, the selection of function-type scaling factor $P_{i}$ lacks specific physical significance and universally applicable methods. The scaling factor is often set as fixed values based on practical engineering requirements, which is convenient but limits its adaptive capability. To address this issue, through analysis of numerous experimental data and scaling factor selection principles, the scaling factor design parameters $P_{i}$ are dynamically adjusted based on e(t) and ec(t) as key parameters, as shown in Equation (19) [36].

(19) $$P=\frac{E_{1}}{|e(t)|(E_{1}+E_{2})+v}+\frac{E_{2}}{|ec(t)|(E_{1}+E_{2})+v}$$

where v is a sufficiently small positive number to ensure the denominator is non-zero. When e(t) and ec(t) approach 0, $P_{i}>1$, at which point $P_{i}=1$. To ensure coordination between input and output variables, let $P_{1}=P_{2}=P_{3}=P_{4}=P$. Substituting this into Equation (18) yields the new function-type scaling factor, as shown in Equation (20).

(20) $$\left\{\begin{array}{l} \alpha (e(t))={\left(\frac{\left|e(t)\right|}{E_{1}}\right)}^{P}+\theta \\ \alpha (ec(t))={\left(\frac{\left|ec(t)\right|}{E_{2}}\right)}^{P}+\theta \\ \beta (e(t),\Delta e(t))=\frac{\alpha (e(t))+\alpha (ec(t))}{2}+\theta \end{array}\right.$$

The VFPID controller constructed with this new scaling factor can be applied to henhouse temperature control systems, achieving rapid adjustment of temperature errors and their rates of change, thereby effectively managing the temperature environment in henhouses.

## 4. Simulation Results and Discussion

**Figure 5:**
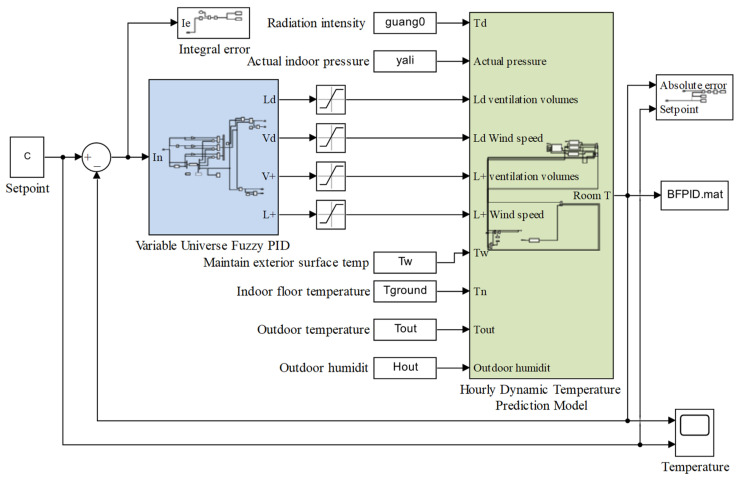
Temperature control model.

To verify the accuracy of the dynamic temperature model and the VFPID control method under CPNPV in the layer house, a temperature control model was built in Simulink, as shown in Figure 5. This study selected measured data from summer, autumn, and winter to compare the mean absolute error (MAE) and root-mean-square error (RMSE) between the simulated values and the actual values, evaluating the precision of the temperature dynamic model and the VFPID temperature control model. Additionally, the VFPID method was compared with the FPID and PID methods.

When constructing the heat exchange model for the layer house, it is necessary not only to collect input data but also to determine the parameters for each module. Some module parameters are determined based on the actual conditions of the experimental henhouse and the characteristics of building materials, making them fixed constants. However, other module parameters need to be dynamically calculated using mathematical methods and adjusted in real time as environmental conditions change. Table 4 summarizes the main parameters of the dynamic temperature model for the layer house, listing the sources and specific values of each parameter.

**Remark** **4.** *This model is only applicable to layer hen houses in northern China that adopt a combined positive- and negative-pressure ventilation system, and it is suitable for the summer, autumn, and winter seasons. Outside of this range, the predictive performance of the model may be affected*.

### 4.1. Verification of Dynamic Temperature Model in Different Seasons

Data for verifying the dynamic temperature model inside the henhouse were selected from 3 June to 12 June 2023, 16 October to 20 October 2023, and 22 January to 27 January 2024, covering three seasons. Four monitoring points were set up inside the henhouse to measure temperature, humidity, and atmospheric pressure, and four sensors were placed inside (and outside) to measure wall surface temperature. Considering that data collected by sensors at different points may vary, the average values of the four sensors measuring temperature, humidity, and atmospheric pressure inside the henhouse, as well as the average values of the four sensors measuring wall surface temperature inside (and outside) the henhouse, were used to represent the actual measured values inside and outside the henhouse. These statistically processed data served as the validation data for the dynamic temperature model of the henhouse for summer, autumn, and winter.

**Figure 6:**
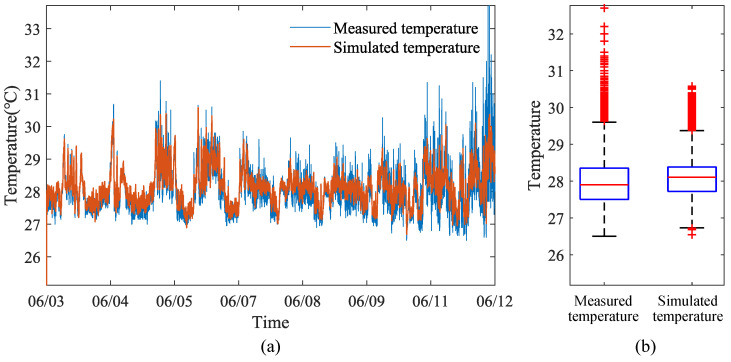
Comparison figure of simulated and actual temperatures inside the layer house during the summer: (**a**) Line figure of temperature variation. (**b**) Box plot of temperature distribution.

**Figure 7:**
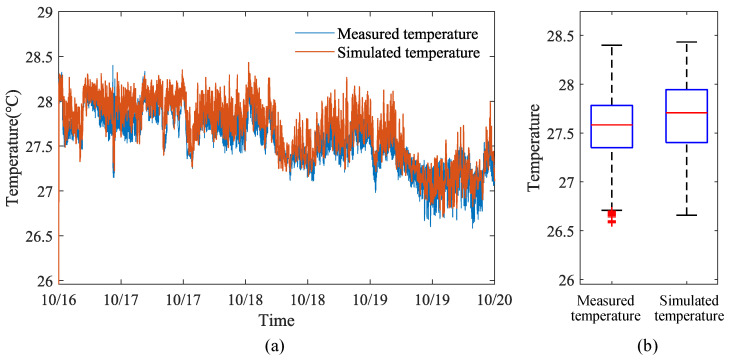
Comparison figure of simulated and actual temperatures inside the layer house during the autumn: (**a**) Line figure of temperature variation. (**b**) Box plot of temperature distribution.

**Figure 8:**
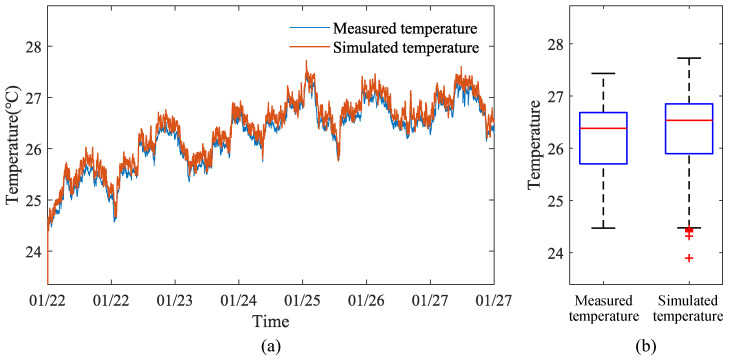
Comparison figure of simulated and actual temperatures inside the layer house during the winter: (**a**) Line figure of temperature variation. (**b**) Box plot of temperature distribution.

The comparison results between the output values of the indoor dynamic temperature model and the measured values are shown in Figure 6, Figure 7 and Figure 8. From the temperature distribution box plots in Figure 6b, Figure 7b and Figure 8b, it can be observed that during the summer, autumn, and winter seasons, the actual and simulated temperature ranges inside the henhouse were 27.50–28.35 °C and 27.72–28.38 °C; 27.35–27.78 °C and 27.40–27.94 °C; and 26.68–25.70 °C and 25.89–26.85 °C, respectively, indicating that the simulated temperature ranges were consistent with the actual measured ranges. The error analysis results between the simulated and measured values for summer, autumn, and winter are shown in Table 5. The model’s R^2^ ranged between 0.79 and 0.88, with the best fit in winter, where R^2^ reached 0.88, the mean absolute error was 0.16 °C, and the maximum error was 0.52 °C.

Therefore, regardless of the season, the dynamic temperature model established in this study demonstrates high accuracy (R^2^ = 0.79–0.88), capable of accurately reflecting temperature changes inside the henhouse even under harsh external environmental conditions. This robust model provides a reliable tool for optimizing henhouse climate control systems and could significantly contribute to improving poultry welfare and production efficiency.

### 4.2. Verification of the VFPID Control Method

The validation of VFPID-based temperature control selected data from 5 June to 6 June 2023, 17 October to 18 October 2023, and 22 January to 23 January 2024, covering three seasons, with two days of data selected for each season for validation.

#### 4.2.1. Simulation Parameter Settings

Inside the henhouse, temperature is significantly influenced by seasonal variations in environmental temperature, with summer exhibiting the highest temperatures and winter the lowest. Consequently, the temperature control model must establish an appropriate target inside the henhouse temperatures for each season and employ distinct PID parameters to precisely regulate ventilation volumes across seasons. Based on practical farming experience, this study designed a seasonal temperature control strategy to maintain stable indoor temperatures. The target indoor temperatures were set at 27.5 °C, 27 °C, and 25.5 °C for summer, autumn, and winter, respectively.

Optimal initial PID algorithm parameters were determined for each season to ensure indoor temperature stability:

Summer: $K_{P}=23$, $K_{I}=15$, $K_{D}=3$.

Autumn: $K_{P}=17$, $K_{I}=13$, $K_{D}=8$.

Winter: $K_{P}=13$, $K_{I}=12$, $K_{D}=4$.

These seasonally adjusted parameters facilitate precise temperature control throughout the year, accommodating the varying environmental conditions characteristic of each season.

#### 4.2.2. Comparative Analysis of the Effects of Different Control Methods

**Figure 9:**
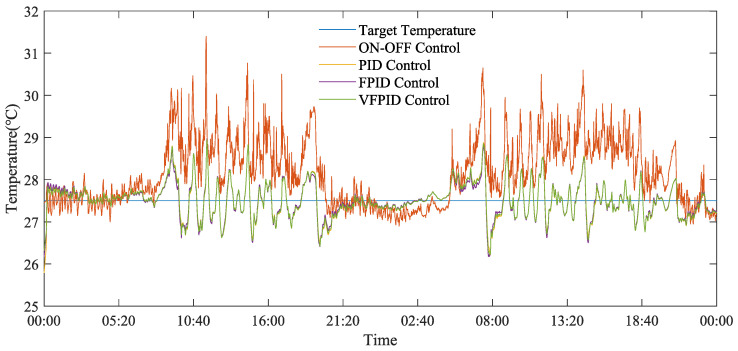
Comparison figure of the effectiveness of different control strategies on indoor temperature regulation during the summer.

**Figure 10:**
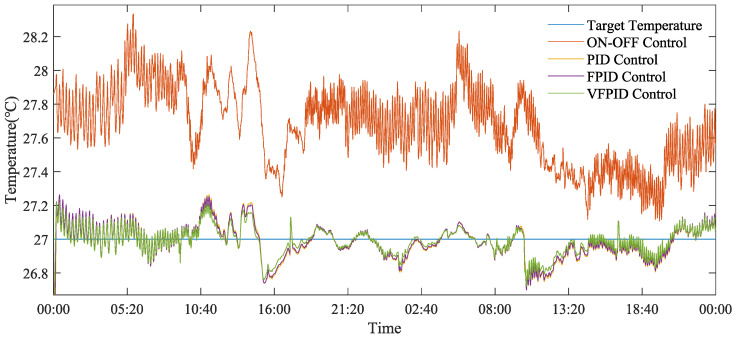
Comparison figure of the effectiveness of different control strategies on indoor temperature regulation during the autumn.

**Figure 11:**
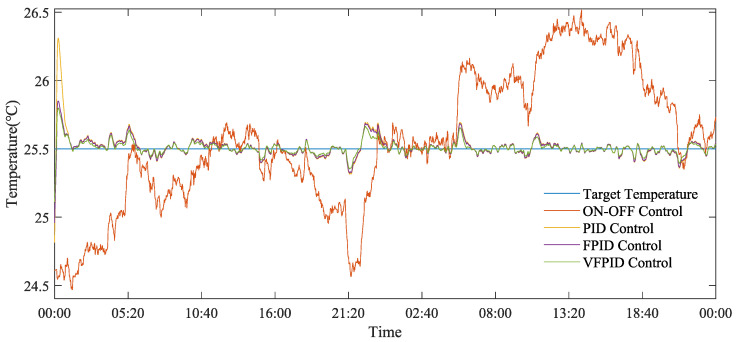
Comparison figure of the effectiveness of different control strategies on indoor temperature regulation during the winter.

Figure 9, Figure 10 and Figure 11 demonstrate the temperature regulation effects of different control methods across three seasons. The on–off control method exhibited large temperature fluctuations in summer, autumn, and winter, with temperature ranges of 26.90–31.40 °C, 27.10–28.33 °C, and 24.47–26.52 °C, respectively, making it difficult to maintain a stable indoor environment. In contrast, the PID, FPID, and VFPID control methods were able to keep the temperature close to the set value, showing significant advantages. Particularly, the VFPID control method achieved peak temperatures of 28.95 °C, 27.22 °C, and 25.79 °C in summer, autumn, and winter, respectively, improving control accuracy by 7.80%, 3.91%, and 2.75% compared with the on–off control method.

In Figure 9, daytime temperature fluctuations are larger in summer, while nighttime fluctuations are smaller, with average errors of 0.82 °C and 0.65 °C during the day and night, respectively, indicating more stable temperature control at night. This difference is mainly attributed to external temperature changes and the physiological adjustments of the layer hens. When external temperature is high, the heat within the henhouse and the heat dissipation from the layer hens increase, making it challenging for the maximum ventilation setting to effectively cool the house, thereby resulting in elevated indoor temperature.

Figure 10 and Figure 11 show better temperature control effects in autumn and winter, with temperatures maintained close to the target value. This is primarily because lower external temperatures require smaller ventilation volumes to achieve the set temperature inside the henhouse.

#### 4.2.3. Quantitative Comparative Analysis

To validate the superiority of the VFPID method developed in this study over other control strategies, a comparative analysis was conducted across different seasons, evaluating the proposed method against traditional PID and FPID approaches. Additionally, a mean oscillation index was introduced to assess the magnitude of temperature fluctuations within the henhouse after regulation by these three control methods [43]. This index is defined by Equation (21).

(21) $${\Delta A}_{oi}=\frac{1}{K^{*}-1}\overset{n}{\underset{t=2}{\sum }}\frac{\left|T(t)-T(t-1)\right|}{T_{av}}*100\%$$

where ${\Delta A}_{oi}$ represents the mean oscillation index for temperature; a lower value indicates more stable system operation; $K^{*}$ denotes the total number of data points; t is the data point index; and $T_{av}$ is the mean temperature.

The experimental errors of different control methods across three seasons are presented in Table 6. Data analysis reveals that seasonal factors significantly influence system stability. The average oscillation amplitudes vary seasonally: 0.155–0.169% in summer, 0.069–0.079% in autumn, and the lowest at 0.041–0.049% in winter. Notably, the VFPID control method consistently achieved the lowest oscillation amplitude across all seasons, demonstrating excellent performance and adaptability. In summer, the response time of the VFPID control method (13.11 min) was 7.28% shorter than that of the traditional PID, while also showing the best performance in terms of overshoot (2.58%), mean absolute error (0.26 °C), and root-mean-square error (0.75 °C). In autumn, the response time (9.01 min) of the VFPID control method was 12.86% shorter than that of the traditional PID, with a mean absolute error of 0.07 °C and a root-mean-square error of 0.61 °C. In winter, although the traditional PID had a slight advantage in response time (7.62 min), the VFPID control method still showed the best performance in terms of overshoot (1.17%), mean absolute error (0.04 °C), and root-mean-square error (0.56 °C). These data indicate that the VFPID method maintains stable control performance under different seasonal conditions, particularly excelling in reducing errors and overshoot, providing strong support for improving the accuracy and stability of the temperature control system.

Thus, whether in summer, autumn, or winter, the traditional PID control method, with fixed parameters, struggles to adapt to environmental changes, resulting in slightly poorer performance regarding overshoot and response speed. While the FPID improves adaptability, it may still lack sufficient response speed under extreme conditions. The VFPID control method proposed in this study excels in regulating temperature within layer houses, effectively adjusting to temperature variations across seasons, particularly demonstrating enhanced performance in winter and autumn. This method introduces a scaling factor that adaptively adjusts the fuzzy universe size based on simulated indoor temperature changes (expanding the universe with increased response and contracting it with decreased response) to achieve more precise temperature control.

### 4.3. Discussion

#### 4.3.1. Impact of External Environment on the Dynamic Temperature Model

In this model, it is assumed that the henhouse environment parameters are uniformly distributed. However, in practice, due to differences in fan positions, ventilation conditions are uneven. When the fans on the east side are activated, the air speed on the west side is significantly lower than that on the east side, leading to significant differences in temperature and humidity in different areas of the henhouse.

As shown in Table 7, the simulated temperature fluctuation range and temperature difference inside the henhouse are the largest in summer, reaching up to 5 °C, particularly between 10:00 and 16:00. This is likely due to the influence of solar radiation outside the henhouse and frequent ventilation inside the henhouse during this period. The enclosure structure absorbs a large amount of heat, causing the indoor temperature to gradually rise, and the heat production from the layer hens also increases. The fan control strategy inside the henhouse operates on a cycle when the temperature exceeds the set value, rather than running all fans at once, leading to instability in the indoor heat. Therefore, during the activation phase, the indoor temperature does not immediately drop to the set value but takes a long time to reach it. As a result, the temperature fluctuation range and temperature difference inside the henhouse are large. In autumn, there is a significant downward trend in indoor temperature from 19 October to 20 October 20 due to the sudden drop in outdoor temperature, leading to a substantial decrease in indoor temperature, with the lowest temperature reaching 26.58 °C. In winter, with the cold weather outside, it is crucial to maintain warmth inside the henhouse, with minimal ventilation to remove harmful gases, resulting in a smaller temperature fluctuation range and an indoor temperature difference of 3 °C.

The largest temperature errors occurred on 3 June from early morning until 4:35 a.m. and from 9:30 p.m. on 4 June to 5:25 a.m. on 5 June, with a maximum error of 2.37 °C. These periods had rainfall, and the cooling pads were not activated, leading to simulated temperatures being higher than the actual values.

#### 4.3.2. Analysis of Seasonal Control Effects

**Figure 12:**
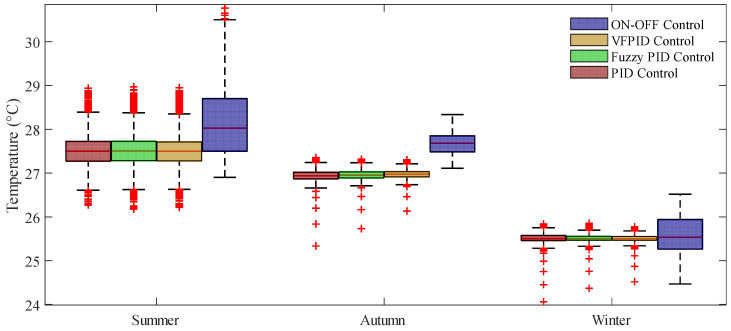
Comparison figure of the temperature control performance of four algorithms.

Figure 12 presents box plots of temperature distribution under different control methods. Under on–off control, the temperature ranges in the three seasons are 27.5–28.7 °C, 27.48–27.85 °C, and 25.26–25.94 °C, respectively, with wide temperature distribution ranges. Under VFPID control, the temperature ranges in the three seasons are 27.28–27.71 °C, 26.91–27.03 °C, and 25.46–25.55 °C, respectively, showing significant improvements in control precision and fluctuation range compared with on–off control. PID control and FPID control have similar temperature medians and interquartile ranges, but the interquartile range for FPID control is slightly smaller than that for PID control, indicating slightly better control performance. For each season, the temperature median for variable universe FPID control is closest to the set value, with the smallest interquartile range and the most concentrated temperature distribution, demonstrating the best control performance.

**Figure 13:**
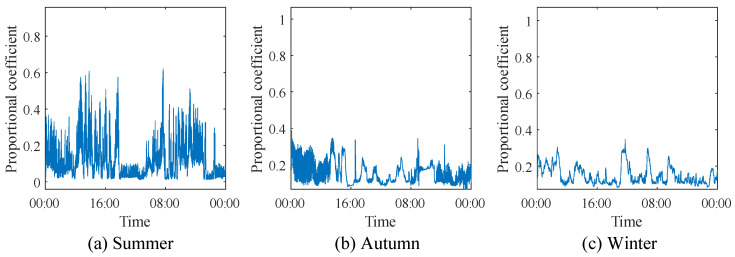
Positive- and negative-pressure coupling coefficient λ.

Figure 13 shows the changes in the positive–negative-pressure coupling coefficient across the three seasons, with average values of 0.3, 0.25, and 0.17, respectively. The data indicate that the curve fluctuation is the greatest in summer, reflecting the high difficulty of temperature regulation inside the henhouse in summer, requiring multiple adjustments of positive-pressure ventilation to reach the set temperature. In contrast, the curve fluctuations in autumn and winter are smaller, indicating that smaller positive-pressure ventilation volumes are sufficient to achieve temperature control.

## 5. Conclusions

Based on energy balance and mass balance equations, this study constructed a dynamic temperature simulation model under CPNPV, accurately reflecting the temperature changes inside the layer house. Based on this model, a VFPID environmental control method for the layer house was proposed, effectively improving the indoor environment quality, control precision, and response speed. The research results indicate the following:

(1) The dynamic temperature simulation model under CPNPV shows a consistent trend with actual values, with mean absolute errors of 0.24 °C in summer, 0.17 °C in autumn, and 0.16 °C in winter, indicating that the model can serve as a foundational model for environmental control in layer houses under combined ventilation.

(2) Comparative analysis of control effects in three different seasons shows that the VFPID control method improves control precision by 20–23.53% and 10.34–22.22% compared with PID and FPID control methods, respectively, indicating superior performance. Additionally, in terms of response time and overshoot, the VFPID controller also exhibits better performance, demonstrating its superior handling of uncertainty and non-linear systems, making temperature control more precise and efficient.

(3) A fuzzy rule-based adaptive control method has been proposed for dynamically adjusting the proportional coefficient λ between positive- and negative-pressure ventilation volumes. This method does not rely on expert knowledge and can automatically adjust the ratio of positive- and negative-pressure ventilation volumes, thereby achieving optimal control results.

The dynamic model and control methodology proposed in this study provide theoretical references for environmental control in livestock farming. Future research should expand to broader environmental tests to fully verify the annual control effects and adaptability inside the layer house. Additionally, combining other environmental parameters such as humidity and carbon dioxide concentrations can further enhance the overall performance of the system, providing more comprehensive environmental control solutions for modern livestock farming.

## Figures and Tables

**Table 1:** Detailed parameters of the environmental monitoring sensor.

The Type of Sensor	Sensor Model	Manufacturer	Accuracy	Measurement Range
Temperature	RS-WS-2D	Shandong Renke Control Technology Co., Ltd., Qingdao, Shandong Province, China	±0.5 °C	−40~+80 °C
Humidity			±3%RH	0~100%RH
Atmospheric pressure	RS-QY-2-4		±0.15 kPa	0~120 kPa
Infrared temperature	RS-WD-HW-I20		±1.5 °C	0~100 °C
Thermal anemometer	GT8911	Shenzhen Jumaoyuan Technology Co., Ltd., Shenzhen, China	±3% ± 0.1 m/s	0.1~30.0 m/s

**Table 2:** Environmental data inside and outside the layer house for three different seasons.

Date	Outdoor Environment			Indoor Environment		
	Weather Conditions	Min/Max Temp °C	Avg. Temp °C	Min/Max Temp °C	Avg. Temp °C	Avg. RH %
3 June 2023	Sunny/Light Rain	14.2/29.1	22.3	27.0/29.1	27.9	48.2
4 June 2023	Cloudy/Cloudy	17.4/25.6	21.7	27.0/30.7	28.9	47.1
5 June 2023	Cloudy/Sunny	15.9/31.1	24.2	27/31.4	28.1	54.6
6 June 2023	Cloudy/Cloudy	18.5/32.2	26.3	26.9/30.7	28.2	41.6
7 June 2023	Cloudy/Sunny	19.3/35.0	26.9	27.1/29.9	27.9	45.4
16 October 2023	Sunny/Sunny	16.5/25.5	20.9	26.6/28.4	27.8	38.0
17 October 2023	Light Rain/Light Rain	13.2/27.4	19.4	27.3/28.3	27.8	45.3
18 October 2023	Cloudy/Cloudy	16.6/26.9	20.1	27.1/28.2	27.6	43.9
19 October 2023	Cloudy/Sunny	14.2/22.1	18.1	26.6/27.9	27.4	34.5
20 October 2023	Sunny/Sunny	12.2/24.3	15.1	26.6/27.6	27.1	35.2
22 January 2024	Sunny/Sunny	−10.5/1.4	−5.7	24.5/25.7	25.2	46.9
23 January 2024	Sunny/Sunny	−8.4/2.8	−3.8	25.4/26.5	25.9	46.5
24 January 2024	Sunny/Cloudy	−4.5/7.7	0.8	25.5/26.8	26.1	44.3
25 January 2024	Cloudy/Cloudy	−2.4/8.7	1.9	25.8/27.4	26.6	44.6
26 January 2024	Sunny/Sunny	−0.3/8.2	2.7	26.2/27.3	26.7	43.4

**Table 3:** $\Delta K_{P}/\Delta K_{I}/\Delta K_{D}/\lambda$ Fuzzy rule table.

ec	e						
	NB	NM	NS	ZO	PS	PM	PB
NB	PB/NB/PS/PB	PB/NB/NS/PM	PM/NM/NB/PS	PS/NM/NB/ZO	PS/NS/NB/NS	ZO/PS/NM/NM	ZO/PS/PS/NB
NM	PB/NB/PS/PM	PB/NB/NS/PS	PM/NM/NB/ZO	PS/NS/NM/NS	PS/NS/NM/NM	ZO/PS/NS/NB	NS/PS/PM/NB
NS	PM/NB/PS/PS	PM/NM/NS/ZO	PM/NS/NM/NS	PS/NS/NM/NM	ZO/PS/NS/NB	NS/PM/NS/NB	NS/PM/PM/NB
ZO	PM/NM/PS/PS	PM/NM/NS/ZO	PS/NS/NS/NS	ZO/PS/NS/NB	NS/PM/NS/NB	NM/PB/NS/NB	NM/PB/PM/NB
PS	PS/NM/PS/PS	PS/NS/PS/ZO	ZO/PS/PS/NS	NS/PM/PS/NM	NS/PM/PS/NB	NM/PB/PS/NB	NM/PB/PS/NB
PM	PS/PS/PB/PM	ZO/PS/NS/PS	NS/PM/PM/ZO	NM/PM/PM/NS	NM/PB/PM/NM	NM/PB/PM/NB	NB/PB/PB/NB
PB	ZO/PS/PB/PB	ZO/PS/PB/PM	NM/PM/PB/PS	NM/PB/PB/ZO	NM/PB/PM/NS	NB/PB/PM/NM	NB/PB/PB/NB

**Table 4:** Main parameters of the temperature dynamic model in layer houses.

Parameter	Source	Value	Unit
NoC	Actual breeding quantity	4.8	Ten thousand birds
VLH	Measurement	6375	m^3^
FAIC	Measurement	1500	m^2^
PMSA	Measurement	920	m^2^
CA	Measurement	1504	m^2^
ERASR	MATLAB calculation	1491	m^2^
RHE-LH	literature [37]	5.4	W/(m^2^·K)
ICC	literature [38,39]	0.64	/
GHTC	literature [40,41]	6	W/m^2^
TCCM	literature [27,42]	0.93	W/(m^2^·K)
TTPIB	literature [27,42]	0.042	W/(m^2^·K)

**Table 5:** Error analysis results between simulated and measured values.

Data Days	Season	Max Error	Min Error	MAE	RMSE	R^2^
10	Summer	2.37	0.00	0.24	0.32	0.79
5	Autumn	0.63	0.00	0.17	0.15	0.81
6	Winter	0.52	0.00	0.16	0.24	0.88

**Table 6:** Error analysis table for different control methods used in the same season.

Season	Control Method	Control Indicators				
		Response Time (min)	${\Delta A}_{oi}$(%)	Overshoot (%)	MAE (°C)	RMSE (°C)
Summer	PID	14.25	0.169	2.87	0.34	0.82
	FPID	13.32	0.162	3.01	0.29	0.79
	VFPID	13.11	0.155	2.58	0.26	0.75
Autumn	PID	10.34	0.079	0.94	0.09	0.71
	FPID	9.96	0.079	1.01	0.09	0.69
	VFPID	9.01	0.069	0.85	0.07	0.61
Winter	PID	7.62	0.049	3.18	0.05	0.59
	FPID	9.40	0.048	1.37	0.05	0.58
	VFPID	8.82	0.041	1.17	0.04	0.56

**Table 7:** Comparison of indoor temperature and external environmental parameters.

Data Days	Season	Indoor Environment			Outdoor Environment		
		Avg. Temp (°C)	Min/Max Temp (°C)	ΔT	Avg. Temp (°C)	Min/Max Temp (°C)	ΔT
10	Summer	27.99	26.50/31.50	5	24.97	14.21/35.03	20.82
5	Autumn	27.55	26.58/28.40	1.82	19.04	9.50/30.06	20.56
6	Winter	26.23	24.47/27.43	3	−0.49	−10.50/8.70	19.2

## Data Availability

The data of this study are available from the corresponding author.

## Abbreviations

CPNPV	Combined Positive- and Negative-Pressure Ventilation
VFPID	Variable Universe Fuzzy PID Control Algorithm
FPID	Fuzzy PID
Min	Minimum
Max	Maximum
Temp	Temperature
Avg	Average
RH	Relative Humidity
NoC	Number of Layer Hens inside the Coop
VLH	Volume of Layer Houses
FAIC	Floor Area Inside the Coop
PMSA	Peripheral Maintenance Structure Area
CA	Ceiling Area
ERASR	Effective Roof Area for Solar Reception
RHE-LH	Radiative Heat Exchange Coefficient of Layer Hens Body Surface
ICC	Irradiance Conversion Coefficient
GHTC	Ground Heat Transfer Coefficient
TCCM	Thermal Conductivity of Cement Mortar
TTPIB	Thermal Transmittance of Polystyrene Insulation Board
MAE	Mean Absolute Error
RMSE	Root-Mean-Square Error
ΔT	Temperature Difference
